# Anti-vascular endothelial growth factor in neovascular age-related macular degeneration – a systematic review of the impact of anti-VEGF on patient outcomes and healthcare systems

**DOI:** 10.1186/s12886-020-01554-2

**Published:** 2020-07-17

**Authors:** Robert P. Finger, Vincent Daien, Bora M. Eldem, James S. Talks, Jean-Francois Korobelnik, Paul Mitchell, Taiji Sakamoto, Tien Yin Wong, Krystallia Pantiri, Joao Carrasco

**Affiliations:** 1 Department of Ophthalmology, University of Bonn, Bonn, Germany; 2 Department of Ophthalmology, Gui de Chauliac Hospital, Montpellier, France; 3 The Save Sight Institute, Sydney Medical School, The University of Sydney, Sydney, NSW Australia; 4 Faculty of Medicine, Ophthalmology Department, Hacettepe University Hospitals, Ankara, Turkey; 5 Department of Ophthalmology, Royal Victoria Infirmary, Newcastle upon Tyne, UK; 6 CHU Bordeaux, Service d’Ophtalmologie, Bordeaux, France; 7 Bordeaux, Inserm, Bordeaux Population Health Research Center, Bordeaux, France; 8 Centre for Vision Research, Westmead Institute for Medical Research, University of Sydney, Sydney, NSW Australia; 9 Department of Ophthalmology, Kagoshima University Graduate School of Medical and Dental Sciences, Kagoshima, Japan; 10 Japan Clinical Retina Study Group (J-CREST Group), Kagoshima, Japan; 11 Singapore Eye Research Institute, Singapore National Eye Centre, Singapore, Singapore; 12 Duke-NUS Medical School, Singapore, Singapore; 13 Pharmerit International, Rotterdam, Netherlands; 14 Bayer Consumer Care AG, Peter Merian-Strasse 84, 4052 Basel, Switzerland

**Keywords:** Age-related macular degeneration, Neovascular, Anti-VEGF, Impact, Systematic review, Visual impairment, Vision-related QoL, Legal blindness, Cost

## Abstract

**Background:**

Systematically review the evidence describing the impact of anti–vascular endothelial growth factor (anti-VEGF) therapy on neovascular age-related macular degeneration (nAMD) patient outcomes and healthcare resource utilization.

**Methods:**

A systematic literature review was completed using Medline and EMBASE for publications prior to July 2018, and proceedings from major ophthalmology conferences (January 2016 to July 2018). The search strategy combined terms for nAMD with terms for anti-VEGF and study design. The review focused on publications describing the impact of anti-VEGF on blindness, visual impairment, vision-related quality of life (VRQoL), mortality, and costs. The search targeted data collected in epidemiological or observational studies to reflect real-world outcomes but also considered modeling-based approaches.

**Results:**

The use of anti-VEGF in clinical practice was associated with significant reduction in the incidence of blindness by nAMD. Population-based analyses reported reduction in incidence among the general population of 47% (9.1 cases/100,000 in 2006 to 4.8 cases/100,000 in 2011). Among patients aged ≥50 years, a reduction of 50% was observed (52.2 cases/100,000 in 2000 to 25.7 cases/100,000 in 2010). In some cases, the odds of decreased vision (defined as decline from normal to moderate, moderate to severe, or severe to blindness) fell by 41% following introduction of anti-VEGF. Patients’ VRQoL improved with treatment, with patients reporting a positive impact shortly after treatment was initiated. Change on National Eye Institute 25-Item Visual Function Questionnaire score from baseline to month 12 ranged from 0.7 to 4.4. Although nAMD patients report signs of depression and anxiety, the evidence suggests that there is no association between the use of anti-VEGF and the prevalence or diagnosis of depression. The introduction of anti-VEGF led to increased overall treatment costs due to replacement of existing less frequently administered treatments (e.g. photodynamic therapy) and increased number of patients treated (prior to anti-VEGF, only ~ 20% of patients were eligible for treatment).

**Conclusions:**

The introduction of anti-VEGF agents has been associated with a positive impact on patient-relevant outcomes, including a significant reduction in incidence of blindness and visual impairment by nAMD. Anti-VEGF agents replaced less-effective treatments, improving patient outcomes and broadening the patient population eligible for treatment.

## Background

Age-related macular degeneration (AMD), the leading cause of irreversible vision impairment and blindness in the developed world, has a profound effect on the quality of life of affected individuals and on healthcare systems [1, 2]. In 2010, AMD was responsible for 5% of the blindness cases registered worldwide [3], and the total healthcare expenditure for managing AMD-related visual impairment surpassed $343 billion [4]. Aging is the greatest risk factor for developing AMD, so its prevalence and the associated socioeconomic burden of the disease are expected to increase significantly as the world’s population ages. By the end of 2020, nearly 196 million people worldwide are expected to have AMD; by 2040, this number will increase to 288 million [5].

Neovascular age-related macular degeneration (nAMD), recognized as the most common late stage of AMD, is responsible for most cases of blindness; nAMD accounts for approximately 10% of AMD cases, but results in 80–90% of legal blindness caused by AMD [6]. nAMD is the only AMD stage for which a specific treatment is presently available: anti–vascular endothelial growth factor (anti-VEGF) agents. Randomized controlled trials established the efficacy and safety of intravitreal aflibercept and intravitreal ranibizumab in nAMD and enabled the licensing of these drugs [7–10]. There is also widespread off-label use of intravitreal bevacizumab in many countries. The introduction of anti-VEGF as a standard treatment in nAMD has led to a great improvement in the prognosis of patients, allowing recovery and maintenance of visual function in most cases [11]. Bloch et al. reported an association between the introduction of anti-VEGF injections and the reduction in blindness in the Danish population [12]. Johnston et al. reported a similar reduction in blindness and sight impairment in the United Kingdom [13]. So far, systematic reviews of anti-VEGF have focused on characterizing clinical outcomes, such as visual acuity (VA), and treatment burden (injection frequency and visits). A broad and comprehensive systematic review on the impact of anti-VEGF beyond VA is lacking.

Patient-centered outcomes and costs associated with the use of anti-VEGF are important outcomes to be studied, in particular from a societal perspective. To this end, this study aims to systematically review the published evidence of the economic impact and the impact on patients’ overall well-being after receiving anti-VEGF therapy for nAMD.

## Methods

### Systematic literature review

A search strategy was developed to identify published research that described the impact of anti-VEGF therapy for nAMD with regard to the following outcomes: vision impairment, legal blindness, vision-related quality of life (VRQoL), risk of mortality, risk of myocardial infarction or stroke, and costs (direct and indirect).

To identify the relevant evidence, we undertook a systematic literature search using Medline and EMBASE (via ProQuest) for publications released prior to July 20, 2018. This search was complemented with a review of proceedings from major ophthalmology conferences (American Academy of Ophthalmology [AAO], Asia-Pacific Academy of Ophthalmology [APAO], Association for Research in Vision and Ophthalmology [ARVO], and European Society of Retina Specialists [EURETINA]) and the International Society for Pharmacoeconomics and Outcomes Research (ISPOR) published from January 2016 to July 2018. To ensure transparency and replicability of the review, we developed a study protocol detailing our approach. All searches were conducted on July 20, 2018.

The search string for ProQuest included both Medical Subject Headings terms and free-text terms. Text terms specifically searched in title or abstract, and included syntax, quotation marks and Boolean operators. Search terms for nAMD were combined with search terms for anti-VEGF therapy and study design. Conference abstracts published earlier than 2016, letters, and editorials were excluded from the search. The search terms for the individual conference databases included terms related to anti-VEGF treatments or the nAMD indication. The search terms used per data source are described in Additional Table 1.

Given the nature of the analysis, an additional step was included. The references in all publications identified in the systematic literature review (SLR) were reviewed to identify any that were not captured by the SLR.

### Selection of studies and data extraction

After all searches were completed, manuscript and conference abstract search hits were imported into a Microsoft Excel (Microsoft; Redmond, WA, USA) file, which was used as the basis to identify relevant publications following a population, intervention, comparator, outcomes, and study-design approach [14]. The results were screened based on title/abstract followed by full-text review by one reviewer after the removal of duplicate publications. Publication eligibility was assessed based on the prespecified inclusion and exclusion criteria provided in Table 1. During both the title/abstract and full-text screening phase, reasons for exclusion were documented according to these predefined criteria. Studies reporting only clinical outcomes, such as change in the number of Early Treatment Diabetic Retinopathy Study (EDTRS) letters, were not included. Data from eligible abstracts and full-text studies were screened and extracted. The reporting of the SLR followed the Preferred Reporting Items for Systematic Reviews and Meta-Analyses (PRISMA) guidelines [24].

**Table 1:** Studies on impact of anti-VEGF treatments on vision-related outcomes

Study	Country/region	Study design	Outcomes measure(s)/definition	Key finding(s)
Bloch et al. [12]	Denmark	Population-based study	BCVA ≤0.1 (20/200) in both eyes; tunnel vision defined as constriction to ≤5 degrees eccentricity or homonymous hemianopia	The incidence rate of legal blindness attributable to AMD in citizens aged > 50 years decreased from 52.2 cases per year per 100,000 in 2000 to 25.7 cases per year per 100,000 in 2010 (50% reduction)
Borooah et al. [15]	Scotland	Population-based study	Blindness (severe sight impairment) defined as: • Snellen VA of < 3/60 with a full visual field • VA between 3/60 and 6/60 with a severe reduction of field of vision (e.g. tunnel vision), or • VA of ≥6/60 but with a very reduced field of vision with their better eye	Incidence of legal blindness due to nAMD per 100,000 population (age-sex standardized): • 2004: 8.5 • 2005: 8.6 • 2006: 9.1 • 2007: 8.8 • 2008: 7.1 • 2009: 6.8 • 2010: 4.9 • 2011: 4.8 Following the introduction of IVTR there were annual decreases in the incidence of blindness. Cases fell to a trough of 4.8/100,000 in 2011 in either eye (drop of 47%)
Bressler et al. [16]	US	Simulation-based study	Legal blindness was defined as VA ≤38 ETDRS letters (comparable to a Snellen equivalent of 20/200) in the better-seeing eye	In case of no treatment, 16,268 individuals would become legally blind over 2 years. The treatment reduced the number of cases of legal blindness by 72% to 4484 individuals.
Campbell et al. [17] (abstract)	US	Cohort-based study	Legally blind, VA 20/200 in better-seeing eye; eyes with incident nAMD and ≥ 12 months of follow-up; two cohorts of patients that are selected to have one cohort before and one after the advent of anti-VEGF therapy	In 2002 (*n* = 84), prevalence of visual impairment (2 years) • 29% (95% CI, 19–39) In 2008 (*n* = 41), prevalence of visual impairment (2 years) • 2% (95% CI, 0–13) Reduction in odds (2002–2008); 95% CI, 59–100
Johnston et al. [13]	UK	Cohort-based study	VA ≤38 ETDRS letters in the better-seeing eye	Percentage of blindness described in the study • 2008: 6.9% • 2009: 3.9% • 2010: 2.0% • 2011: 2.4% Cumulative incidence of new blindness at follow-up, with significant reductions in the rates between year cohorts • At 1 year: 5.1% • At 2 years: 8.6% • At 3 years: 12.0% • At 4 years: 15.6%
Keenan et al. [18]	UK	Cohort-based study	0–24 letters correspond to eligibility for full CVI; 25–39 letters to eligibility for partial CVI	The proportion of patients in the study eligible at baseline for full or partial CVI decreased from 13.8% in 2008 to 7.1% in 2012 (*P* = 0.04).
Minassian et al. [19]	UK	Simulation-based study	Blindness defined as VA of < 6/60 in the better seeing eye	Blindness was expected to increase from 90,254 in 2010 to 120,452 in 2020, assuming that 75% of those eligible patients are treated with the approved anti-VEGF
Mitchell et al. [20]	Australia	Simulation-based study	BCVA < 6/60 (approximate ETDRS letter score ≤ 38) in the better-seeing eye	Without treatment, 2246 individuals would become legally blind over 2 years. With treatment, the incidence of blindness was reduced by 68–72%
Rostron et al. [21]	UK	Population-based study	Sight impairment and severe visual impairment used on the UK certificate of visual impairment & incidence of visual impairment certification due to AMD^a^	After the introduction of ranibizumab in 2008, the incidence of visual impairment certification due to nAMD dropped from 225 per million population in 2005 to 137 per million in 2010 after 2008
Skaat et al. [22]	Israel	Population-based study	BCVA of < 1/60 or central visual field ≤10 degrees in the less impaired eye; incidence of certified blind population in Israel due to AMD and other causes	The incidence of newly registered legal blindness at the end of the studied decade was half that at the beginning, declining from 33.8 per 100,000 population in 1999 to 16.6 per 100,000 population in 2008
Sloan et al. [23]	US	Cohort-based study	Sight impairment based on the ICD-9-CM codes for severe vision loss and blindness decrease in vision, vision loss/blindness	Vision loss or blindness was 2.04% in the 2 years following a first exudative AMD diagnosis; the introduction of anti-VEGF therapy reduced vision loss or blindness by 46% (OR, 0.54; 95% CI, 0.47–0.63)

^a^Sight impairment: Corrected Snellen visual acuity of 3/60 or 6/60 with full fields, corrected Snellen visual acuity of ≤6/24 with moderate constriction of visual field, or corrected Snellen visual acuity of ≥6/18 with gross visual field defects. Severe sight impairment: corrected Snellen visual acuity < 3/60, corrected Snellen visual acuity between 3/60 and 6/60 with very contracted visual fields, or corrected Snellen visual acuity of ≥6/60 with a very contracted visual field especially in the lower part of the field

*Abbreviations*: *AMD* age-related macular degeneration, *BCVA* best corrected visual acuity, *CI* confidence interval, *CVI* certificate of visual impairment, *ETDRS* Early Treatment Diabetic Retinopathy Study, *ICD-9-CM* International Classification of Diseases, Ninth Revision, Clinical Modification, *IVTR* intravitreal ranibizumab treatment, *nAMD* neovascular AMD, *OR* odds ratio, *UK* United Kingdom, *US* United States, *VA* visual acuity, *VEGF* vascular endothelial growth factor

## Results

### SLR results

**Fig. 1:**
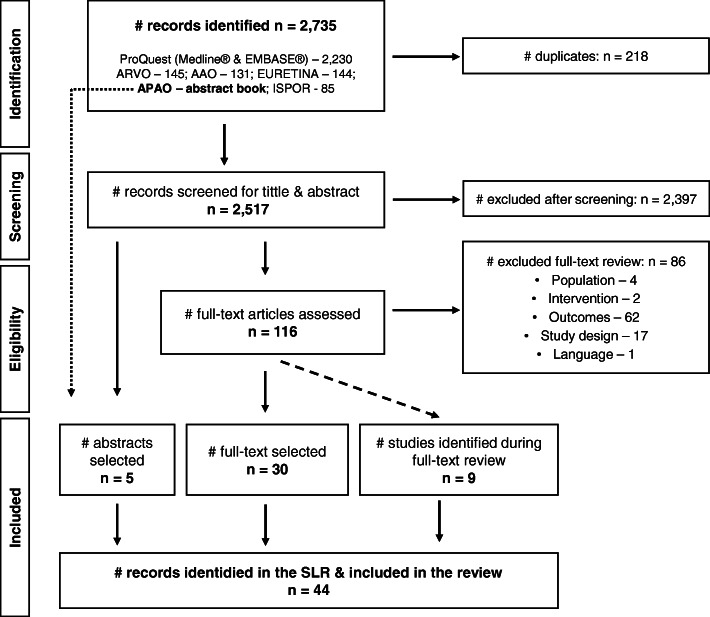
PRISMA flow diagram. AAO, American Academy of Ophthalmology; ARVO, Association for Research in Vision and Ophthalmology; APAO, Asia-Pacific Academy of Ophthalmology; EURETINA, European Society of Retina Specialists; ISPOR, International Society for Pharmacoeconomics and Outcomes Research; PRISMA, Preferred Reporting Items for Systematic Reviews and Meta-Analyses; SLR, systematic literature review. The dotted line represents a manual search of the APAO abstract book

The SLR identified 2735 records. The database search identified 2230 publications (manuscripts) via Medline and EMBASE (via ProQuest). An additional 505 publications (conference abstracts) were identified from conference proceedings (ARVO, AAO, EURETINA, APAO, and ISPOR). The search for conference proceedings included the manual review of three abstract books from APAO, from which one study was identified and selected to be included in the analysis (Fig. 1).

After removing 218 duplicates, the title and abstract of 2517 publications were screened for eligibility before moving to full-text screening. After screening, 2397 publications were excluded based on title and abstract. Of the remaining 120 publications, four were abstracts from conference proceedings and 116 were manuscripts. The four abstracts were selected to be included in the analysis. Of the 116 manuscripts, 86 were excluded after review. Studies were excluded primarily because they did not report data on the outcomes of interest (62) or because of study design (17). Thus, 30 manuscripts moved forward to full-text review. During the full-text review, the reference lists from the 30 manuscripts was revised using the same approach, and nine additional articles were identified. At the end of the review, 44 publications were selected (39 manuscripts and five conference abstracts) for extraction (Fig. 1).

The final list of publications included in this review consists of 11 studies describing the impact of anti-VEGF therapy on vision-related outcomes, including blindness, other vision impairment outcomes, and the ability to drive; four studies (five publications) on the patients’ overall quality of life (QoL); six studies on the depression or anxiety in patients receiving anti-VEGF treatment; nine studies on the risk for mortality, myocardial infarction, or stroke); and 13 studies on the impact of anti-VEGF therapy on costs.

### Impact on vision-related outcomes

Our review identified 11 publications reporting on vision-related outcomes (ten manuscripts and one conference abstract) [12, 13, 15–23]. After review, and based on the data reported, the vision-related outcomes studies were divided into three subcategories: 1) blindness, 2) visual impairment, and 3) ability to drive. A summary overview of the studies is provided in Table 1.

#### Blindness

All 11 publications reported data on blindness, although the definition of blindness was not the same for all studies (Table 1). The study design also varied across the studies and included four population-based studies, four cohort-based studies, and three simulation-based studies with hypothetical cohorts.

Among the population-based studies, Bloch et al. and Skaat et al. estimated the evolution of incidence rates of legal blindness from AMD using national blindness registry data from Denmark and Israel [12, 22]. Both studies describe outcomes before and after anti-VEGF therapies were introduced in the local clinical practice, and both reported a nearly 50% drop in the incidence rate of legal blindness by AMD since anti-VEGF was introduced [12, 22]. Rostron et al. reported the incidence rate of UK Visual Impairment Certification due to nAMD in the Leeds metropolitan area [21]. The analysis shows that the incidence of new registrations in the area dropped by nearly 50% (from 225 per million population to 137 per million) after the introduction of anti-VEGF in the UK’s National Health Service (NHS). Borooah et al. used blind certifications recorded by the Royal National Institute of Blind People in South East Scotland to estimate the impact of anti-VEGF on blindness attributable to nAMD [15]. The study reports a 47% drop in the number of blind certification cases between 2006 (date of introduction of anti-VEGF therapy) and 2011.

For the cohort studies, Sloan et al. used US Medicare data to analyze vision loss or blindness in the first 2 years following a new diagnosis of nAMD and found a 46% reduction in vision loss or blindness [23]. Johnston et al. and Keenan et al. used electronic medical records from UK hospitals (14 individual sites and one NHS Trust, respectively) to estimate the impact of anti-VEGF on vision-related outcomes [13, 18]. The research done by Johnston et al. reported a significant reduction on the cumulative incidence of new blindness [13], whereas Keenan et al. reported a 49% reduction in patients eligible for full or partial certificate of visual impairment [18]. The study by Campbell et al. (conference abstract) reports data on two small cohorts of patients: one each before and after introduction of anti-VEGF [17]. The study demonstrated a 95% reduction in the incidence of legal blindness [17]. Due to the limited sample size and non-availability of full text, this result should be interpreted with caution.

The review also identified three simulation studies [16, 19, 20]. Based on US, UK, and Australian perspectives, each study simulated the evolution of a hypothetical cohort of nAMD patients and estimated the number of blindness cases potentially avoided due to the introduction of anti-VEGF in clinical practice [16, 19, 20]. Each study used local data to reflect the age and sex of the targeted population and other key variables (e.g. mortality, incidence/prevalence of nAMD) and estimated the impact attributed to anti-VEGF treatment by modeling the efficacy reported in the randomized clinical trial. Both Bressler et al. (US) and Mitchell et al. (Australia) estimated that the use of anti-VEGF could reduce the number of legal blindness cases due to nAMD by 70% [16, 20]. Minassian et al. (UK) assessed the impact of anti-VEGF treatment on the prevalence of sight loss attributable to nAMD against expected demographic changes and concluded that the potential benefit of anti-VEGF would be outweighed by the aging effect and that the overall rate of blindness could increase in the future [19]. There are differences in the modeling approaches used by Bressler et al., Mitchell et al., and Minassian et al. in their analysis. The authors consider different approaches to model changes in demographics, efficacy of the anti-VEGF, and coverage. Additionally, the baseline characteristics of the patients are different. The different assumptions and the different modeling approaches, in particular how demographic changes are modeled, could justify the different conclusions.

#### Visual impairment and ability to drive

Cohort studies by Sloan et al. and Campbell et al. also reported the impact of anti-VEGF on other vision-related outcomes, such as degree of visual impairment [17, 23]. Sloan et al. reported that the odds of decreased vision (defined as decline from normal to moderate, moderate to severe, or severe to blindness, where moderate, severe, and blindness is defined according to the International Classification of Diseases, Ninth Revision, Clinical Modification code) fell by 41% after introduction of anti-VEGF therapy (odds ratio, 0.59; 95% confidence interval, 0.52–0.68) [23]. Campbell et al. reported that moderate and mild impairment (defined as 20/80 and 20/40 in the better-seeing eye) was reduced by 78 and 70%, respectively, after introduction of anti-VEGF therapies [17]. The simulation studies by Bressler et al. and Mitchell et al. also estimated that visual impairment among patients with nAMD (defined as a letter score of 68 or lower, thus including blindness) could be reduced by 28 to 37% with the use of anti-VEGF [16, 20].

Keenan et al. was the only study that reported on the impact of anti-VEGF therapy on the ability to drive [18]. In this study, the proportion of nAMD patients suitable for driving (defined as VA in the better eye ≥70 EDTRS letters) increased from 27% in 2008 (before anti-VEGF) to 51.4% in 2012 (*P* < 0.0001).

### Impact on VRQoL

#### Impact of anti-VEGF therapy on patient’s QoL

Our review identified five publications from four studies that explored the impact of anti-VEGF on patients’ VRQoL [25–28]. The impact on VRQoL was assessed using the National Eye Institute Visual Function Questionnaire (NEI VFQ-25) in three of the studies [26–28] and the Impact of Vision Impairment (IVI) questionnaire in one study [25]. Key findings per study are provided in Table 2.

**Table 2:** Studies on impact of anti-VEGF treatments on vision-related QoL

Study	Study design	Outcomes measure(s)/definition	Key finding(s)	
*Impact of anti-VEGF therapy on patient’s quality of life (four studies; five publications)*				
Zhu et al. [28] (abstract)	Prospective open-label clinical trial	Patients’ VRQoL using the NEI-VFQ-25 at 6, 12,18, and 24 months	Improvement in VRQoL at 6, 12, 18, and 24 months: Composite score: 4.5 ± 9.2/4.4 ± 11.8/5.6 ± 11.2/4.6 ± 12.4 o Good responder: 4.4 ± 8.9/6.8 ± 10.1 o Poor responder: 4.6 ± 9.6/2.5 ± 12.7 o Mental health: 6.2 ± 13.3/4.3 ± 15.2 o Driving: 1.7 ± 19.8/− 2.1 ± 16.9	
Inoue et al. [27]	Observational non-interventional study	NEI VFQ-25 scores preoperatively and postoperatively at 3 months/12 months	Score at baseline: Composite score: 72.3 o Mental health: 68.4 o Driving: 69	Score at 3/12 months: • 75.8/78.5 o 77.2/78.6 o 70.1/69
			IVR treatment resulted in a higher postoperative NEI VFQ-25 score Improved VA at 12 months was associated with a greater improvement in NEI VFQ-25	
Finger et al. [25]	Observational, non-interventional study	The VRQoL at 6 and 12 months was measured by the IVI using its three subscales: Accessing information, Mobility, and Emotional well-being	Score at baseline: • Accessing information: − 0.54 ± 2.33 • Mobility: − 0.82 ± 2.68 • Emotional well-being: − 0.97 ± 2.68	Score at 6 months/12 months • –0.67 ± 2.07/− 0.55 ± 2.35 • –0.93 ± 2.53/− 0.69 ± 2.69 • –1.17 ± 2.68/− 1.11 ± 3.06
Finger et al. [26]	Observational, non-interventional study	Patients’ VRQoL using the NEI-VFQ at 12 months	Improvements in VRQoL at 12 months: + 0.73 ± 0.37	
*Depression and anxiety after anti-VEGF therapy for nAMD (six studies)*				
Casten et al. [29]	Observational, non-interventional study	• PHQ-9 rating severity of depressive symptoms at baseline and at 3 months • Subjective opinion of how helpful injections and obstacles to treatment	• At 3 months, 20% of patients had clinically significant depressive symptoms (mean [SD] PHQ-9 score, 6.8 [1.6]) • Compared with non-depressed patients, depressed patients had a greater decline in vision over 3 months • Depression was unrelated to changes in NEI-VFQ scores or obstacles to treatment	
Cooley et al. [30] (abstract)	Prospective observational study	PSS, CES-D, IVI; Relationships among changes in VA, IVI, PSS, and CES-D were analyzed using linear regression	• Greater social support at initiation of anti-VEGF treatment was associated with reduced depression at follow-up • Decrease in self-reported visual functioning was related to higher stress level at follow-up, whereas VA change was not	
Lee et al. [31]	Cross-sectional study	Prevalence of depression using geriatric depression scale	• The prevalence of depression: 26.2% with AMD; it was suggested that age was the most important factor associated with depression in AMD • With older age, the severity of depression also increases	
Segal et al. [32]	Observational non-interventional study	Pre-procedural anxiety using VASA	Positive correlation between increased preprocedural anxiety and perceived pain	
		Post-procedural pain using VAS	Correlation between procedure and perceived pain in intravitreal injections	
Senra et al. [33]	Observational, cross-sectional study; mixed methods	Qualitative data on patients’ experience with treatment	56% of patients reported anxiety related to anti-VEGF treatment. The main sources of anxiety: fear of going blind due to intravitreal injections and concerns about treatment effectiveness, rather than pain	
		Standardized validated questionnaires to quantify clinically significant levels of anxiety (HADS-Α), depression (HADS-D), and posttraumatic stress (patients) (IES-R), cognitive function (MMSE) and caregivers’ burden	• 17% of patients showed clinical levels of anxiety • 12% showed clinical levels of depression • Depression levels, but not anxiety, were significantly higher in patients who received ≤3 injections compared with patients who received 4–12 injections and patients who received > 12 injections	
Sloan et al. [23]	Longitudinal	Number of patients newly diagnosed with depression during the follow-up period (measure or method not stated)	A new diagnosis of depression during the follow-up period was found to be 2%; there was no statistical difference between those who had anti-VEGF treatment and those who did not	
		Need for admission to a long-term care facility	Receipt of anti-VEGF therapy was associated with a 19% lower probability of entry into a long-term care facility	

*Abbreviations*: *AMD* age-related macular degeneration, *CES-D* Center for Epidemiological Studies Depression scale, *HADS* Hospital Anxiety and Depression Scale, *IES-R* Impact of Events Scale-Revised, *IVI* Impact of Vision Impairment scale, *IVR* intravitreal injection of ranibizumab, *MMSE* Mini-Mental State Examination, *NEI VFQ-25* National Eye Institute Visual Function Questionnaire, *PHQ-9* Patient Health Questionnaire 9, *PSS* Perceived Stress Scale, *SD* standard deviation, *VAS* visual analogue scale, *VASA* visual analogue scale for anxiety, *VEGF* Vascular endothelial growth factor, *VRQoL* vision-related quality of life

The NEI VFQ-25 contains one general health item, and 25 questions measuring dimensions of self-reported vision-targeted health status that are considered most important for individuals with any chronic eye disease such as nAMD [34]. Questions are categorized into eleven vision-related subscales on: general vision (one question), ocular pain (two questions), near-vision and distance-vision activities (three questions each), social functioning (two questions), mental health (four questions), role difficulties (two questions), dependency and driving (three questions each), and color vision and peripheral vision (one question each). Each subscale is transformed on a 0–100 scale, with higher scores indicating better subjective function. The NEI VFQ-25 composite score is calculated as the mean score of all vision-related subscales, and excluding the general health item [34].

The IVI questionnaire contains 28 items with three to four active response options that employed Likert scaling, ranging from *not at all* to *a lot* [35, 36]. Items 1 to 15 have an additional response: *don’t do this for other reasons*. These items form three specific subscales: Reading and Accessing Information, Mobility and Independence, and Emotional Well-being [35, 36].

All the studies using NEI-VFQ-25 reported an improvement in patients’ VRQoL after 12 months of treatment. The change in total composite score of NEI VFQ-25 from baseline to month 12 ranged from 0.7 to 4.4 [26–28]. This improvement was associated with the improved vision in the treatment eyes. Inoue et al. and Zhu et al. showed improvement in the VRQoL of nAMD patients after a shorter period of treatment; mean change in total composite score on the NEI VFQ-25 was 3.5 at 3 months of treatment and 4.5 at 6 months of treatment. In general, all studies saw improvement in patients’ VRQoL after start of intravitreal treatment (NEI VFQ-25 scores improved at reported time points compared with baseline; *P* < 0.05 for all) [25, 27, 28].

Finger et al., Inoue et al., and Zhu et al. also assessed the impact of anti-VEGF treatment on patients’ mental health and emotional well-being. Both dimensions were improved from baseline to last follow-up at 12 months [25, 27, 28].

#### Depression and anxiety while receiving anti-VEGF therapy for nAMD

Our review identified six studies that assessed the impact of anti-VEGF therapies on VRQoL (Table 2) [23, 29–33].

In the pilot study by Casten et al., depression in nAMD patients receiving anti-VEGF therapy was assessed using the Patient Health Questionnaire 9 (PHQ-9) [29]. PHQ-9 rates the severity of depressive symptoms based on the Diagnostic and Statistical Manual of Mental Disorders (Fourth Edition) criteria for depression [37]. Scores range from 0 to 27; a score greater than 5 is clinically significant. In this report, the authors calculated the prevalence rate of depression to be 20% among patients receiving anti-VEGF treatments and reported that these were slightly lower than in previous studies conducted before the widespread use of anti-VEGF treatments. In addition, depressed patients were found to have a greater decline in vision despite treatment compared with non-depressed patients [37]. Interesting, depression was unrelated to changes in NEI-VFQ scores [37].

Lee et al. analyzed 107 Korean patients with nAMD treated with anti-VEGF using the Geriatric Depression Scale [38] and found a 26.2% prevalence of depression, which is consistent with published rates before anti-VEGF treatments became widely available [31]. It was further suggested that age is the most important factor associated with depression in AMD, with older age, being associated with more severe depression [31].

The incidence of a first diagnosis of depression was analyzed among Medicare beneficiaries in the United States in a retrospective study of patients diagnosed with nAMD [23]. Overall, only 2.0% of the patient population studied received first diagnosis of depression during the 2-year follow-up period and the study reported no significant differences in the incidence of depression in patients who received anti-VEGF treatments compared with those who did not. However, the study did find that patients receiving anti-VEGF therapy had a 19% lower probability of being admitted into a long-term care facility.

Two studies explored patients’ anxiety and depression levels associated with receiving intravitreal injections [32, 33]. The first was a prospective observational study which was conducted in Israel. This study found a significant correlation between patients’ anxiety levels experienced before the injection and pain experienced when receiving the injection [32], with 25% of patients reported high levels of anxiety (score ≥ 6 on a scale of 0–10) measured by a visual analogue scale. The second study [33] was a prospective study in the United Kingdom and assessed the patient experiences while receiving anti-VEGF treatment for nAMD, measuring clinically significant levels of depression, anxiety, and post-traumatic stress using the Hospital Anxiety and Depression Scale (HADS) [39] and the Impact of Events Scale–Revised [40]. The study showed that 56% of patients reported anxiety related to the intravitreal injection. The main sources of anxiety included fear of going blind because of having an injection in the eye and worry about treatment effectiveness, rather than fear of pain associated with the injection. The questionnaires indicated that 17 and 12% of patients had clinical levels of anxiety and depression, respectively. The study found that the level of depressive symptoms, but not of anxiety, was significantly higher in patients who received up to three injections than in patients who received 4–12 injections and in patients who received > 12 injections (analysis of variance, *P* = 0.027 and *P* = 0.001, respectively). They also found that the frequency of clinical levels of depression (HADS-depression score ≥ 8) decreased with increasing numbers of injections. The authors concluded that patients who receive anti-VEGF therapy often experience with some level of anxiety, despite familiarity with the process from previous injections. On the other hand, depression seemed to be more frequent in patients at early stages of anti-VEGF treatment [33] and as anti-VEGF treatment proceeds, patients can become more optimistic about treatment success and disease stabilization [33].

Another prospective study (conference abstract) examined the determinants of perceived stress and depression after anti-VEGF treatment in 114 people with AMD [30]. Overall, the study found that greater social support at initiation of anti-VEGF treatment was associated with reduced depression at follow-up; a decrease in self-reported visual functioning was related to higher stress level at follow-up, whereas objectively measured VA change was not.

### Impact on costs

Our review identified 13 studies that reported on the costs associated with the introduction of anti-VEGF treatments for the treatment of nAMD [41–53].

Six of the 13 studies used claims data to assess the evolution of treatment costs and reached similar conclusions. There was a trend of increasing costs due to the introduction of anti-VEGF therapy and a simultaneous decrease in costs for other treatment options for nAMD patients [41, 48–52]. Kume et al. reported that the treatment cost for AMD per 10,000 individuals in Japan increased approximately 9-fold over 9 years (2005 to 2013) [48]. Rosenfeld et al. reported that the annual total cost for anti-VEGF drugs only for US patients in the Medicare/Medicaid population treated with anti-VEGF (intravitreal aflibercept, bevacizumab, ranibizumab) more than doubled between 2008 and 2015, primarily due to an increased number of nAMD patients being treated with anti-VEGFs [52]. Key findings per study are provided in Table 3.

**Table 3:** Studies on impact of anti-VEGF treatment on costs and resource use

Study	Study population	Study design	Outcomes measure(s)/definition	Key finding(s)
*Trend of increasing costs*				
Campbell et al. [41]	Canada; Ontario Health Insurance Plan	Claims analysis	Total drug costs (anti-VEGF) for Ontario and Canada (2005–2007).	Increase of 8-fold between September 2005 and November 2007 This rapid increase preceded the availability of ranibizumab, strongly suggesting that off-label intravitreal injection of bevacizumab has been highly prevalent
Coleman et al. [51] (abstract)	US Medicare beneficiaries (5% sample, *n* = 6290)	Claims analysis	Total eye-related Medicare costs per patient for 5-year study period (1995–1999) based on reimbursed eye-related professional fees; costs of treatment before introduction of PDT and anti-VEGF	Mean (SD): 2371 (2449); median $1607
Day et al. [49]	US Medicare beneficiaries	Claims analysis	Distribution of mean Medicare payments for nAMD (1994, 2000, 2006)	Increase of costs largely due to anti-VEGF; dramatic rise between 2004 and 2006 then plateaued Diagnosis more than doubled between 1994 and 2006
Kume et al. [48]	Japanese patients with employee health insurance	Claims analysis	Medical expenses per 10,000 patients (2005–2013)	Increase of 9-fold over 9 years, from $1530 to $13,700 Increase of AMD patients by 300%
Qualls et al. [50]	US Medicare beneficiaries	Claims analysis	Direct medical costs per patient/per case, 1 year before and after the index year (2004–2008)	Costs rose from 2004 to 2006, then plateaued Costs in 2008 cohort were 50% higher than in 2004 Costs attributable to anti-VEGF injections: 4% in 2004; 75% in 2008 cohort
Rosenfeld et al. [52]	US Medicare and Medicaid	Claims analysis	Total drug costs (anti-VEGF) for Medicare/Medicaid population (2008–2015)	Increase of 2-fold over 8 years, due to an increased number of nAMD patients being treated with anti-VEGF
*Cost savings*				
Hanemoto et al. [42]	Patients and their private caregivers from one hospital in Japan	Cross-sectional survey	Mean estimated total annual caregiving costs	90,327.11 ¥ total annual costs Treatment via T&E rather than PRN reduced number of hospital visits, a reduction in caregiver burden (time, costs, and emotional impact)
Windsor et al. [43]	US Medicare beneficiaries	Cohort	Medicare reimbursement rate; actual Medicare spending (2008–2015)	$9.0 billion of government savings by using OCT guided anti-VEGF therapy
*Cost estimates reported per country*				
Campbell et al. [41]	Canada; Ontario Health Insurance Plan	Claims analysis	Total drug costs (anti-VEGF) for Ontario and Canada (2005–2007)	Projected total cost (of anti-VEGF drugs) in Canada (2007): • Bevacizumab: $2,769,000 • Ranibizumab: $180,000,000
Fabiano et al. [44]	Patients from five hospitals in Italy	Clinical database	Mean per-capita costs of treatment and specialist (2016)	2536 € (treated < 1 year) 1839 € (treated > 1 year)
Kiss et al. [53] (abstract)	US Patients (data source not reported)	Claims analysis	Mean annual costs per patient (2011–2015)	Treatment = naïve patients • First year with intravitreal aflibercept vs ranibizumab: $10,417 vs $11,032; • First 2 years: $15,410 vs. $15,393 Previously treated patients • First year with intravitreal aflibercept and ranibizumab: $11,521 vs $11,589 • First 2 years: $19,202 vs $18,548
Matamoros et al. [45]	French patients who are members of Association DMLA/Retina France	Cross-sectional survey	Mean cost per year per patient/net annual cost for patient (2012–2013)	1741 € (SD 3397 €, range 0–3176)
Qualls et al. [50]	US Medicare beneficiaries	Claims analysis	Direct medical costs per patient/per case, 1 year before and after the index year (2004–2008)	Costs rose between 2004 and 2006, then plateaued. Costs in 2008 cohort were 50% higher than in 2004. Costs attributable to anti-VEGF injections: 4% in 2004; 75% in 2008 cohort
Rosenfeld et al. [52]	US Medicare and Medicaid	Claims analysis	Total drug costs (anti-VEGF) for Medicare/Medicaid population (2008–2015)	Total annual drug costs in 2008: • Intravitreal aflibercept: not applicable • Bevacizumab: $35,502,851 (583,351 doses) • Ranibizumab: $704,066,862 (327,663 doses) Total annual drug costs in 2015: • Intravitreal Aflibercept: $1,738,642,274 (836,425 doses) • Bevacizumab: $89,488,151 (1,225,348 doses) • Ranibizumab: $1,133,896,626 (542,820 doses)
Schmidt et al. [47]	Patients of largest public ophthalmologic clinic in Switzerland	Claims analysis	Total healthcare costs per patient/per month, directly attributed to anti-VEGF therapy (2006–2014)	2186.98 CHF (95% CI: 1184.58 to 3189.38) In the subgroup of patients with AMD, the costs for ophthalmologic treatment sank by 97.23 CHF/year (95% CI, 985.38–790.92; *P* = 0.829)
Shalaby et al. [46]	Patients from UK NHS ophthalmological units (189 requests; 95.8% responses)	Cross-sectional request	Estimated annual costs of anti-VEGF drugs (incl. VAT) (2015)	Total: £539,764,992 Bevacizumab only: £729,500

*Abbreviations*: *AMD* age-related macular degeneration, *CHF* Swiss franc, *CI* confidence interval, *DMLA* La Dégénérescence Maculaire Liée à l’Age, *nAMD* neovascular AMD, *OCT* optical coherence tomography, *PDT* photodynamic therapy, *PRN* as needed, *SD* standard deviation, *T&E* treat-and-extend, *UK* United Kingdom, *VAT* value-added tax, *VEGF* vascular endothelial growth factor

Day et al. found that the number of Medicare beneficiaries diagnosed with nAMD more than doubled in 2006 compared with a 1994 cohort; they reported that this higher number was associated with the increase in treatment costs and that it had a large impact on the overall cost of nAMD treatment [49].

Two studies reported on the cost savings associated with the use of anti-VEGF treatments in nAMD patients [42, 43]. Windsor et al. concluded that the use of optical coherence tomography (OCT)-guided anti-VEGF therapy (i.e. investment to develop OCT imaging, reimbursement of OCT imaging, savings from fewer anti-VEGF injections) from 2008 to 2015 generated $9 billion in savings [43]. The total patient savings by using OCT-guided anti-VEGF therapy (based on a 20% copay for anti-VEGF therapy, and avoiding 17.7 million IVIs from 2008 to 2015) could amount to $2.2 billion. Hanemoto et al. showed cost savings associated with the implementation of a proactive treat-and-extend (T&E) regimen when compared with as-needed dosing, mostly due to a reduction in hospital visits [42]. The T&E regimen was also associated with reduced caregiver burden. This includes time, out-of-pocket costs, emotional impact of accompanying patients to the hospital, and mean estimated annual productivity loss (T&E was associated with a mean difference in cost of $82,059.49; *P* = 0.00284; corresponding to $679.57 after the first year).

All other identified studies provided annual cost estimates for anti-VEGF treatments (intravitreal aflibercept, bevacizumab, ranibizumab) across various European countries, Canada, and the United States (Table 3).

## Discussion

This systematic review highlights the considerable improvement in nAMD patients’ outcomes related to anti-VEGF treatment as well as the associated increased healthcare resource utilization since anti-VEGF was introduced into clinical practice. The evidence identified in the SLR established a clear temporal association between the introduction of anti-VEGF treatment and the reduction in the incidence of blindness by nAMD. Prior to the availability of real-world data, modeling exercises using randomized clinical trial data postulated a significant reduction in the incidence of blindness by nAMD of up to 70% based on anti-VEGF treatment [16, 19, 20]. The findings from population-based studies [12, 15, 22] support the modeling estimates, albeit with smaller magnitudes in the reduction in blindness incidence of up to 50%. This might be due to the slowing of visual loss with anti-VEGF treatment, which has been shown to be effective in maintaining long-term vision in patients [15, 54].

The evidence identified in the SLR also supports the conclusion that treatment with anti-VEGF is associated with an improvement in patients’ VRQoL. The SLR also highlights the lack of detailed information on how anti-VEGF treatment impacts key aspects of patients’ functionality. All identified studies used VRQoL instruments that might not capture the overall general health spectrum as generic QoL measures do. Therefore, further research into the QoL of nAMD patients undergoing anti-VEGF treatment, taking a longitudinal and holistic approach, is warranted. Another aspect that warrants further research is the impact of anti-VEGF on a patient’s mental health. The limited evidence identified in the SLR suggests that there is no association between the use of anti-VEGF treatments and the prevalence or diagnosis of depression [23, 29, 31]. Patients with nAMD frequently report signs of depression and anxiety, especially early in treatment with anti-VEGF [33], but it remains unclear how this might impact treatment adherence, persistence, and outcomes.

The introduction of anti-VEGF drugs has led to an increase in the overall AMD treatment costs, as exemplified by Japan, where the cost of treating AMD increased by 9-fold between 2005 and 2013 [48]. The increase was driven primarily by two elements. First, anti-VEGF therapy replaced other, less costly treatment options administered less often and for shorter periods of time, and second, the number of patients receiving treatment for nAMD increased substantially (from 0.084 to 0.26% of the population). Parallel with the increase in nAMD patients treated, the different healthcare systems registered a significant increase in the number of doses administered per patient (i.e. treatment intensity). Between 2008 and 2015, the Medicare system registered a 16-fold increase in the number of individual doses of anti-VEGF administered (14.8 million doses in 2015) [52]. The evolution of clinical practice also led to efficiency gains based on OCT-guided anti-VEGF therapy and T&E regimens leading to resource use optimization and substantial savings [43]. However, this was not sufficient to offset the increase in costs associated with the current broad, systematic use of anti-VEGF therapy as the preferred treatment option for nAMD.

To our knowledge, this is the first study to investigate the societal impact of anti-VEGF treatment for nAMD and cover visual outcomes, QoL, and economic cost. The principal strength of this SLR is the use of systematic methods to standardize the selection of studies and the extraction of data. Relevant publications referenced in any identified reviews and meta-analyses were also screened to ensure a comprehensive review. The review excluded studies reporting only clinical outcomes, such as improvement on change in visual acuity (ETDRS letters). Thus, the review focused only on studies reporting meaningful outcomes to patients, such as visual impairment, blindness, and VRQoL. Through this review process, we identified many studies reporting patients’ experiences with receiving anti-VEGF treatments for nAMD. Due to the qualitative research methods in these studies and consequently the not-quantifiable outcomes reported, those findings were not covered in this review.

There are some limitations to the present study. The most important are the lack of useful comparative data, heterogeneity of the included studies, and lack of information on specific subpopulations, such as different ethnic groups. This can be largely attributed to the diversity and inconsistency of reporting formats. Synchronizing reporting and measurement formats is a relevant issue in all secondary research. The identified studies are heterogeneous because of variability in patient characteristics, baseline VA, disease severity, outcome measures employed, and duration of follow-up. Finally, the findings of our review should be interpreted with caution as most studies were conducted in mainly white populations in North America and Europe.

## Conclusion

Anti-VEGF treatment for nAMD has been used in clinical practice for more than a decade, but our understanding of its patient-relevant benefits and societal impacts are still incomplete. The introduction of anti-VEGF therapies led to a significant increase in the number of nAMD patients receiving treatment and required healthcare systems to increase the resources allocated to treat nAMD. We can establish a clear link between the introduction of anti-VEGF treatment and a significant reduction in the incidence of blindness by nAMD, which comes at considerable cost to healthcare systems. However, there is limited evidence on the impact of anti-VEGF treatment on other patient-related outcomes.

## Supplementary information

**Additional file 1: Table 1.** Study eligibility criteria.

## Data Availability

Not applicable

## Abbreviations

AAO: American Academy of Ophthalmology
AFL: Intravitreal aflibercept
AMD: Age-related macular degeneration
APAO: Asia-Pacific Academy of Ophthalmology
ARVO: Association for Research in Vision and Ophthalmology
BCVA: Best corrected visual acuity
CES-D: Center for Epidemiological Studies Depression scale
CI: Confidence interval
CVI: Certificate of visual impairment
ETDRS: Early Treatment Diabetic Retinopathy Study
HADS: Hospital Anxiety and Depression Scale
ICD-9-CM: International Classification of Diseases, Ninth Revision, Clinical Modification
IES-R: Impact of Events Scale-Revised
ISPOR: International Society for Pharmacoeconomics and Outcomes Research
IVI: Impact of Vision Impairment scale
IVR: Intravitreal injection of ranibizumab
IVTR: Intravitreal ranibizumab treatment
MMSE: Mini-Mental State Examination
nAMD: Neovascular age-related macular degeneration
NEI VFQ 25: National Eye Institute Visual Function Questionnaire
NHS: National Health Service
OCT: Optical coherence tomography
OR: Odds ratio
PCV: Polypoidal choroidal vasculopathy
PDT: Photodynamic therapy
PHQ-9: Patient Health Questionnaire 9
PRISMA: Preferred Reporting Items for Systematic Reviews and Meta-Analyses
PRN: Pro re nata
PSS: Perceived Stress Scale
pts.: Patients
RAP: Retinal angiomatous proliferation
RBZ: Ranibizumab
SD: Standard deviation
SLR: Systematic literature review
T&E: Treat-and-extend
VA: Visual acuity
VAS: Visual analogue scale
VASA: Visual analogue scale for anxiety
VAT: Value-added tax
VEGF: Vascular endothelial growth factor
VRQoL: Vision-related quality of life
