# Supplementary material for: Anti-vascular endothelial growth factor in neovascular age-related macular degeneration – a systematic review of the impact of anti-VEGF on patient outcomes and healthcare systems
Source: BMC Ophthalmol. 2020 Jul 17;20:294. doi: 10.1186/s12886-020-01554-2 (PMC7368708; doi:10.1186/s12886-020-01554-2)
Supplement: Supplementary file 1 — Additional file 1: Table 1. Study eligibility criteria. [file 12886_2020_1554_MOESM1_ESM.docx]

# Additional file

# Additional Table 1. Study eligibility criteria

| Criteria | Inclusion criteria | Exclusion criteria |
| --- | --- | --- |
| Population | - Wet age-related macular degeneration (including the subtype PCV) - Studies with mixed ophthalmology indications will only be included if results for wet AMD patients are reported separately - Adults | - Non-humans - Specific subtypes of nAMD, such as RAP - Other ophthalmology indications - No separately reported results for wet AMD population |
| Intervention/comparator | All anti-VEGF treatments as a class and the following individual treatments:   - Intravitreal aflibercept - Ranibizumab - Bevacizumab - Pegaptanib | Other treatments than those listed in the inclusion criteria |
| Outcomes | Outcomes of interest include:  Impact of anti-VEGF therapy on   - Visual impairment (i.e. number of cases of visual impairment avoided) - Legal blindness (i.e. number of cases of legal blindness avoided) - QoL of the patients (including general well-being, mental and physical health, patient-reported outcomes) - Psychosocial and psychological impact (e.g. depression, anxiety) - Mortality - Direct and indirect costs and resource utilization | - Impact of anti-VEGF treatment on visual acuity defined by change (improvement or impairment) in letters - Comparative effectiveness - Comparative costs - Cost-effectiveness - Qualitative outcomes - Other outcomes than those listed in the inclusion criteria |
| Study design | - Observational studies - Non-interventional study - Modeling-based studies - Simulation-based studies | - Randomized studies - Interventional study - Qualitative studies (e.g, patient surveys, interviews) - Systematic reviews* - Meta-analysis - Editorials - Letters to the editor |
| Language | - English (full-text or abstracts^†^) - Full text with English abstract, which can be translated with an online translation tool | - Other languages |
| Location | - All | - None |

*References of systematic reviews will be consulted. ^†^Full text with English abstract that can be translated with an online translation tool.

AMD, age-related macular degeneration; nAMD, neovascular AMD; PCV, polypoidal choroidal vasculopathy; QoL, quality of life; RAP, retinal angiomatous proliferation; VEGF, vascular endothelial growth factor.
